# Crystal structure of 3-carbamo­thio­yl­pyridinium thio­cyanate

**DOI:** 10.1107/S2056989014026437

**Published:** 2015-01-01

**Authors:** Hasna Bouchareb, Mhamed Boudraa, Sofiane Bouacida, Hocine Merazig, El Hossain Chtoun

**Affiliations:** a Unité de Recherche de Chimie de l’Environnement et Moléculaire Structurale, CHEMS, Université Constantine 1, 25000 , Algeria; b Département Sciences de la Matière, Faculté des Sciences Exactes et Sciences de la Nature et de la Vie, Université Oum El Bouaghi, Algeria; c Université Abdelmalek Essaadi, Faculté des Sciences, BP 2121 M’Hannech II, 93002 Tétouan, Morroco

**Keywords:** crystal structure, 3-carbamo­thio­ylpyridinium cation, thio­cyanate anion, N—H⋯S hydrogen bonding

## Abstract

In the cation of the title salt, C_6_H_7_N_2_S^+^·SCN^−^, the C=S bond is oriented *trans* with respect to the C—C=N fragment in the pyridine ring. The planes of the aromatic ring and the thio­amide fragment of the cation make a dihedral angle of 38.31 (4)°. In the crystal, the components are linked by N—H⋯S and N—H⋯N, hydrogen bonds, forming a two-dimensional network parallel to (10-1).

## Related literature   

For isomeric thio­nicotinamide structures, see: Downie *et al.* (1972 ▸); Form *et al.* (1973 ▸); Colleter & Gadret (1967 ▸). For a related structure, see: Sharif *et al.* (2009 ▸). For the structural inter­est of thio­nicotinamides, see: Fonari *et al.* (2007 ▸).
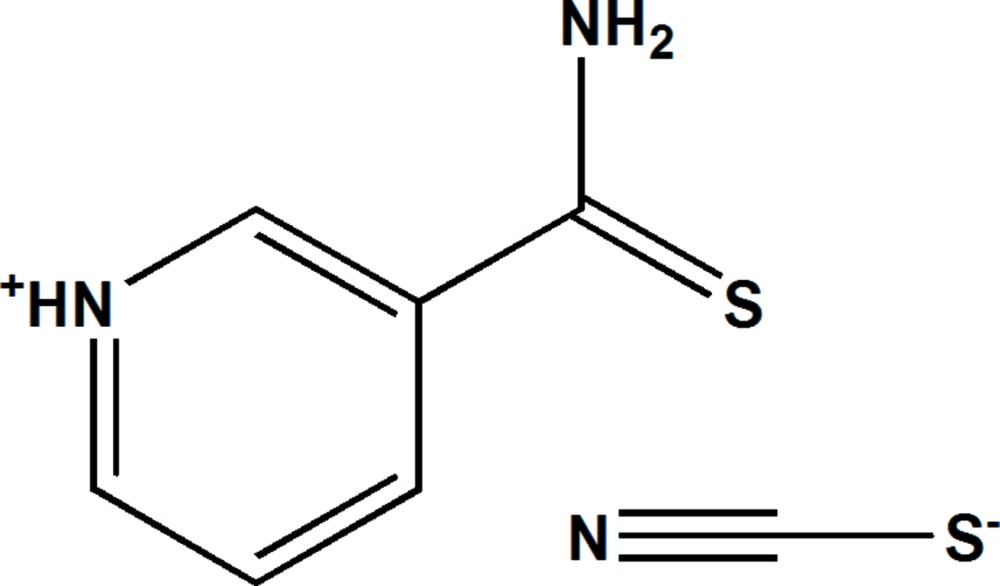


## Experimental   

### Crystal data  

C_6_H_7_N_2_S^+^·CNS^−^
*M* *_r_* = 197.28
Monoclinic, 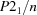

*a* = 7.2495 (2) Å
*b* = 9.3759 (3) Å
*c* = 13.5949 (3) Å
β = 94.454 (1)°
*V* = 921.26 (4) Å^3^
*Z* = 4
Mo *K*α radiation
μ = 0.52 mm^−1^
*T* = 295 K
0.2 × 0.16 × 0.1 mm

### Data collection  

Bruker APEXII diffractometer
Absorption correction: multi-scan (*SADABS*; Sheldrick, 2002 ▸) *T*_min_ = 0.679, *T*_max_ = 0.746
12686 measured reflections
3313 independent reflections
2563 reflections with *I* > 2σ(*I*)
*R*_int_ = 0.023

### Refinement  

*R*[*F* ^2^ > 2σ(*F* ^2^)] = 0.045
*wR*(*F* ^2^) = 0.140
*S* = 1.04
3313 reflections
109 parameters
H-atom parameters constrained
Δρ_max_ = 0.58 e Å^−3^
Δρ_min_ = −0.26 e Å^−3^

### 

Data collection: *APEX2* (Bruker, 2011 ▸); cell refinement: *SAINT* (Bruker, 2011 ▸); data reduction: *SAINT*; program(s) used to solve structure: *SIR2002* (Burla *et al.*, 2005 ▸); program(s) used to refine structure: *SHELXL97* (Sheldrick, 2008 ▸); molecular graphics: *ORTEP-3 for Windows* (Farrugia, 2012 ▸) and *DIAMOND* (Brandenburg & Berndt, 2001 ▸); software used to prepare material for publication: *WinGX* (Farrugia, 2012 ▸).

## Supplementary Material

Crystal structure: contains datablock(s) I. DOI: 10.1107/S2056989014026437/bq2397sup1.cif

Structure factors: contains datablock(s) I. DOI: 10.1107/S2056989014026437/bq2397Isup2.hkl

Supporting information file. DOI: 10.1107/S2056989014026437/bq2397Isup3.cml

**Fig. 1.**
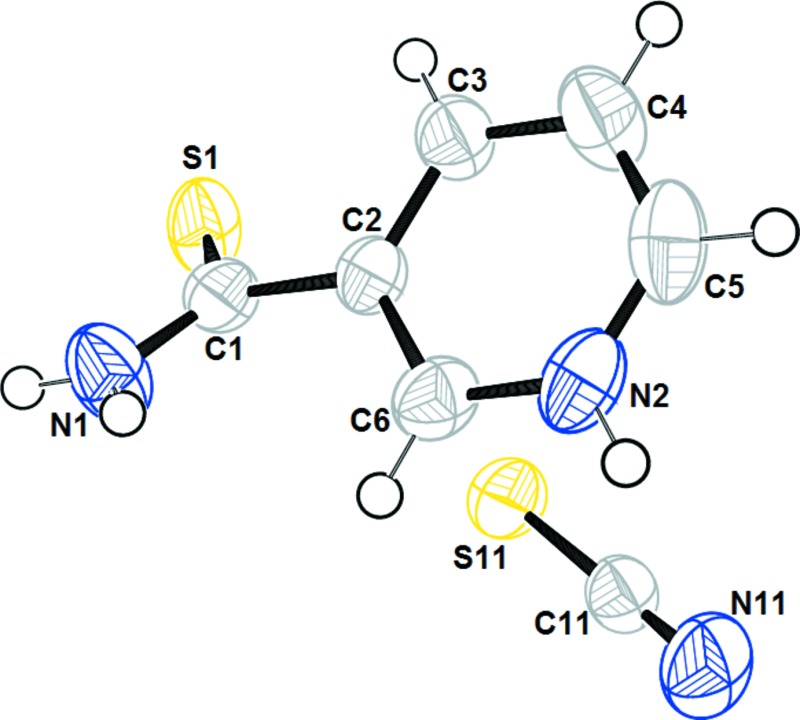
The molecular structure of, (I), with displacement ellipsoids drawn at the 50% probability level. H atoms are represented as small spheres of arbitrary radii.

**Fig. 2.**
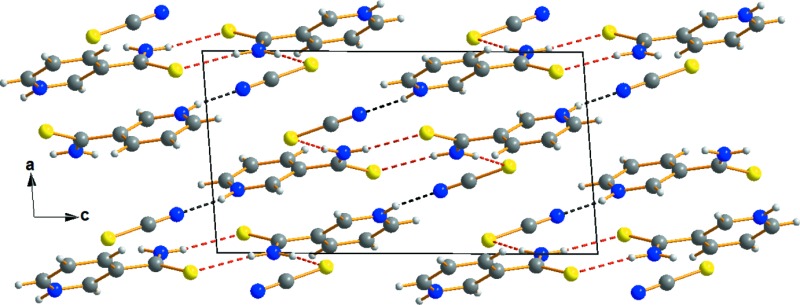
Packing diagram of (I) viewed along the b axis showing hydrogen bond as dashed lines [N—H···S in red and N—H···N in black]

. DOI: 10.1107/S2056989014026437/bq2397fig1.tif
The mol­ecular structure of, (I), with displacement ellipsoids drawn at the 50% probability level. H atoms are represented as small spheres of arbitrary radii.

b . DOI: 10.1107/S2056989014026437/bq2397fig2.tif
Packing diagram of (I) viewed along the *b* axis showing hydrogen bond as dashed lines [N—H⋯S in red and N—H⋯N in black]

CCDC reference: 1037011

Additional supporting information:  crystallographic information; 3D view; checkCIF report

## Figures and Tables

**Table 1:** Hydrogen-bond geometry (, )

*D*H*A*	*D*H	H*A*	*D* *A*	*D*H*A*
N1H1*A*S11^i^	0.86	2.54	3.3450(15)	156
N1H1*B*S1^ii^	0.86	2.55	3.3975(14)	171
N2H2N11^iii^	0.86	1.88	2.709(2)	162

Symmetry codes: (i) 
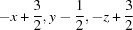
; (ii) 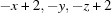; (iii) 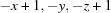.

## supplementary crystallographic information

### S1. Comment

There are three isomeric thionicotinamides of general formula C_6_H_6_N_2_S, 2-thioamidopyridine (Downie *et al.*, 1972), 3-thioamidopyridine (Form *et al.*, 1973) and 4-thioamidopyridine (Colleter & Gadret, 1967), then three possible coordination sites. The structural interest to these compounds has centered on the parameters of the thioamide group and the consequent electron arrangement within the group (Fonari *et al.*, 2007). The thioamide group and the pyridine ring are not coplanar.

In the title compound, (I), the asymmetric unit contains one 3-3-carbamothioylpyridinium and one thiocyanate ions. The molecular geometry and the atom-numbering scheme are shown in Fig 1. In the cation moiety, the C=S bond is oriented *trans* with respect to the C—C—N fragment in the pyridine ring. The aromatic ring and the thioamide fragment of the thionicotinamide molecule make a dihedral angle of 38.31 (4)° similar to that found in 3-carbamothioylpyridinium iodide (*trans*) (30.02 (3)°) (Sharif *et al.*, 2009) and 3-thioamido-pyridine (*cis*) (33.76 (7)°) (Form *et al.* 1973). The crystal packing is stabilized by weak N—H···N and N—H···S hydrogen bonds forming a two-dimensional network (Fig. 2).

### S2. Experimental

3-Thionicotinamide (690 mg, 0.5 mmol) was added dropwise to a solution of KSCN (48.6 mg, 0.5 mmol) in water/ethanol (10 ml/10 ml). The mixture was then refluxed with stirring for 3 h and the resulting solution was left to stand at room temperature. After several days, single crystals suitable for X-ray diffraction were obtained.

### S3. Refinement

Approximate positions for all H atoms were first obtained from the difference electron density map. However, the H atoms were situated into idealized positions and the H-atoms have been refined within the riding atom approximation. The applied constraints were as follow: C—H = 0.93 Å and N—H = 0.86 Å with *U*_iso_ = 1.2*U*_eq_(C or N).

### Crystal data

**Table:**

C_6_H_7_N_2_S^+^·CNS^−^	*F*(000) = 408
*M**_r_* = 197.28	*D*_x_ = 1.422 Mg m^−^^3^
Monoclinic, *P*2_1_/*n*	Mo *K*α radiation, λ = 0.71073 Å
*a* = 7.2495 (2) Å	Cell parameters from 5267 reflections
*b* = 9.3759 (3) Å	θ = 2.6–32.1°
*c* = 13.5949 (3) Å	µ = 0.52 mm^−^^1^
β = 94.454 (1)°	*T* = 295 K
*V* = 921.26 (4) Å^3^	Prism, colorless
*Z* = 4	0.2 × 0.16 × 0.1 mm

### Data collection

**Table:**

Bruker APEXII diffractometer	3313 independent reflections
Radiation source: sealed tube	2563 reflections with *I* > 2σ(*I*)
Graphite monochromator	*R*_int_ = 0.023
φ and ω scans	θ_max_ = 32.5°, θ_min_ = 2.6°
Absorption correction: multi-scan (*SADABS*; Sheldrick, 2002)	*h* = −10→10
*T*_min_ = 0.679, *T*_max_ = 0.746	*k* = −14→14
12686 measured reflections	*l* = −20→20

### Refinement

**Table:**

Refinement on *F*^2^	Primary atom site location: structure-invariant direct methods
Least-squares matrix: full	Secondary atom site location: difference Fourier map
*R*[*F*^2^ > 2σ(*F*^2^)] = 0.045	Hydrogen site location: inferred from neighbouring sites
*wR*(*F*^2^) = 0.140	H-atom parameters constrained
*S* = 1.04	*w* = 1/[σ^2^(*F*_o_^2^) + (0.0808*P*)^2^ + 0.1946*P*] where *P* = (*F*_o_^2^ + 2*F*_c_^2^)/3
3313 reflections	(Δ/σ)_max_ < 0.001
109 parameters	Δρ_max_ = 0.58 e Å^−^^3^
0 restraints	Δρ_min_ = −0.26 e Å^−^^3^

### Special details

**Table:**

Geometry. All e.s.d.'s (except the e.s.d. in the dihedral angle between two l.s. planes) are estimated using the full covariance matrix. The cell e.s.d.'s are taken into account individually in the estimation of e.s.d.'s in distances, angles and torsion angles; correlations between e.s.d.'s in cell parameters are only used when they are defined by crystal symmetry. An approximate (isotropic) treatment of cell e.s.d.'s is used for estimating e.s.d.'s involving l.s. planes.
Refinement. Refinement of *F*^2^ against ALL reflections. The weighted *R*-factor *wR* and goodness of fit *S* are based on *F*^2^, conventional *R*-factors *R* are based on *F*, with *F* set to zero for negative *F*^2^. The threshold expression of *F*^2^ > σ(*F*^2^) is used only for calculating *R*-factors(gt) *etc*. and is not relevant to the choice of reflections for refinement. *R*-factors based on *F*^2^ are statistically about twice as large as those based on *F*, and *R*- factors based on ALL data will be even larger.

### Fractional atomic coordinates and isotropic or equivalent isotropic displacement parameters (Å^2^)

**Table:**

	*x*	*y*	*z*	*U*_iso_*/*U*_eq_	
C1	0.9430 (2)	0.08889 (15)	0.83615 (10)	0.0351 (3)	
C2	0.91545 (18)	0.13467 (15)	0.73124 (9)	0.0322 (3)	
C3	0.9619 (2)	0.27153 (17)	0.70337 (11)	0.0401 (3)	
H3	1.0114	0.336	0.7503	0.048*	
C4	0.9342 (3)	0.3117 (2)	0.60509 (13)	0.0536 (4)	
H4	0.9675	0.4025	0.5854	0.064*	
C5	0.8576 (3)	0.2168 (2)	0.53738 (12)	0.0552 (5)	
H5	0.8365	0.2434	0.4716	0.066*	
C6	0.8395 (2)	0.04215 (18)	0.65938 (10)	0.0392 (3)	
H6	0.8072	−0.0501	0.6764	0.047*	
C11	0.3607 (2)	0.10281 (16)	0.67580 (11)	0.0383 (3)	
N1	0.9976 (2)	−0.04356 (15)	0.85163 (10)	0.0492 (3)	
H1A	1.0153	−0.0983	0.8025	0.059*	
H1B	1.0156	−0.0754	0.9109	0.059*	
N2	0.81305 (19)	0.08559 (17)	0.56595 (9)	0.0464 (3)	
H2	0.7655	0.027	0.5224	0.056*	
N11	0.3114 (3)	0.0569 (2)	0.59942 (12)	0.0642 (5)	
S1	0.90233 (7)	0.20284 (5)	0.92615 (3)	0.04789 (14)	
S11	0.43232 (7)	0.16631 (5)	0.78372 (3)	0.04908 (14)	

### Atomic displacement parameters (Å^2^)

**Table:**

	*U*^11^	*U*^22^	*U*^33^	*U*^12^	*U*^13^	*U*^23^
C1	0.0405 (7)	0.0356 (6)	0.0286 (5)	−0.0036 (5)	−0.0012 (5)	−0.0032 (5)
C2	0.0340 (6)	0.0364 (6)	0.0257 (5)	0.0005 (5)	0.0004 (4)	−0.0029 (4)
C3	0.0481 (8)	0.0358 (7)	0.0358 (6)	0.0008 (6)	−0.0001 (6)	−0.0007 (5)
C4	0.0724 (12)	0.0460 (9)	0.0427 (8)	0.0019 (8)	0.0056 (8)	0.0102 (7)
C5	0.0638 (11)	0.0713 (12)	0.0299 (7)	0.0139 (9)	0.0000 (7)	0.0074 (7)
C6	0.0421 (7)	0.0435 (7)	0.0316 (6)	−0.0039 (6)	0.0007 (5)	−0.0066 (5)
C11	0.0440 (7)	0.0352 (7)	0.0356 (6)	0.0024 (6)	0.0029 (5)	0.0006 (5)
N1	0.0781 (10)	0.0370 (7)	0.0323 (6)	0.0049 (7)	0.0022 (6)	0.0002 (5)
N2	0.0484 (7)	0.0609 (9)	0.0286 (5)	0.0024 (6)	−0.0043 (5)	−0.0102 (5)
N11	0.0865 (12)	0.0625 (10)	0.0419 (7)	0.0034 (9)	−0.0070 (7)	−0.0116 (7)
S1	0.0704 (3)	0.0440 (2)	0.02793 (18)	0.01027 (18)	−0.00476 (16)	−0.00570 (13)
S11	0.0598 (3)	0.0521 (3)	0.0344 (2)	−0.00810 (19)	−0.00268 (16)	−0.00319 (15)

### Geometric parameters (Å, º)

**Table:**

C1—N1	1.3154 (19)	C5—N2	1.337 (3)
C1—C2	1.4879 (18)	C5—H5	0.93
C1—S1	1.6677 (14)	C6—N2	1.3334 (19)
C2—C3	1.387 (2)	C6—H6	0.93
C2—C6	1.3879 (19)	C11—N11	1.155 (2)
C3—C4	1.388 (2)	C11—S11	1.6303 (16)
C3—H3	0.93	N1—H1A	0.86
C4—C5	1.367 (3)	N1—H1B	0.86
C4—H4	0.93	N2—H2	0.86
			
N1—C1—C2	116.23 (12)	N2—C5—H5	120.1
N1—C1—S1	123.75 (11)	C4—C5—H5	120.1
C2—C1—S1	120.02 (11)	N2—C6—C2	119.97 (15)
C3—C2—C6	118.52 (13)	N2—C6—H6	120
C3—C2—C1	120.76 (12)	C2—C6—H6	120
C6—C2—C1	120.71 (13)	N11—C11—S11	179.35 (17)
C2—C3—C4	119.60 (15)	C1—N1—H1A	120
C2—C3—H3	120.2	C1—N1—H1B	120
C4—C3—H3	120.2	H1A—N1—H1B	120
C5—C4—C3	119.51 (17)	C6—N2—C5	122.51 (14)
C5—C4—H4	120.2	C6—N2—H2	118.7
C3—C4—H4	120.2	C5—N2—H2	118.7
N2—C5—C4	119.88 (15)		
			
N1—C1—C2—C3	−142.88 (16)	C2—C3—C4—C5	1.4 (3)
S1—C1—C2—C3	37.84 (19)	C3—C4—C5—N2	−1.1 (3)
N1—C1—C2—C6	38.3 (2)	C3—C2—C6—N2	0.1 (2)
S1—C1—C2—C6	−140.98 (13)	C1—C2—C6—N2	178.93 (13)
C6—C2—C3—C4	−0.9 (2)	C2—C6—N2—C5	0.2 (2)
C1—C2—C3—C4	−179.75 (15)	C4—C5—N2—C6	0.3 (3)

### Hydrogen-bond geometry (Å, º)

**Table:**

*D*—H···*A*	*D*—H	H···*A*	*D*···*A*	*D*—H···*A*
N1—H1*A*···S11^i^	0.86	2.54	3.3450 (15)	156
N1—H1*B*···S1^ii^	0.86	2.55	3.3975 (14)	171
N2—H2···N11^iii^	0.86	1.88	2.709 (2)	162

Symmetry codes: (i) −*x*+3/2, *y*−1/2, −*z*+3/2; (ii) −*x*+2, −*y*, −*z*+2; (iii) −*x*+1, −*y*, −*z*+1.
