# Supplementary figures and images for: Crystal structure of 3-carbamo­thio­yl­pyridinium thio­cyanate
Source: Acta Crystallogr E Crystallogr Commun. 2015 Jan 1;71(Pt 1):o30–1. doi: 10.1107/S2056989014026437 (PMC4331844; doi:10.1107/S2056989014026437)

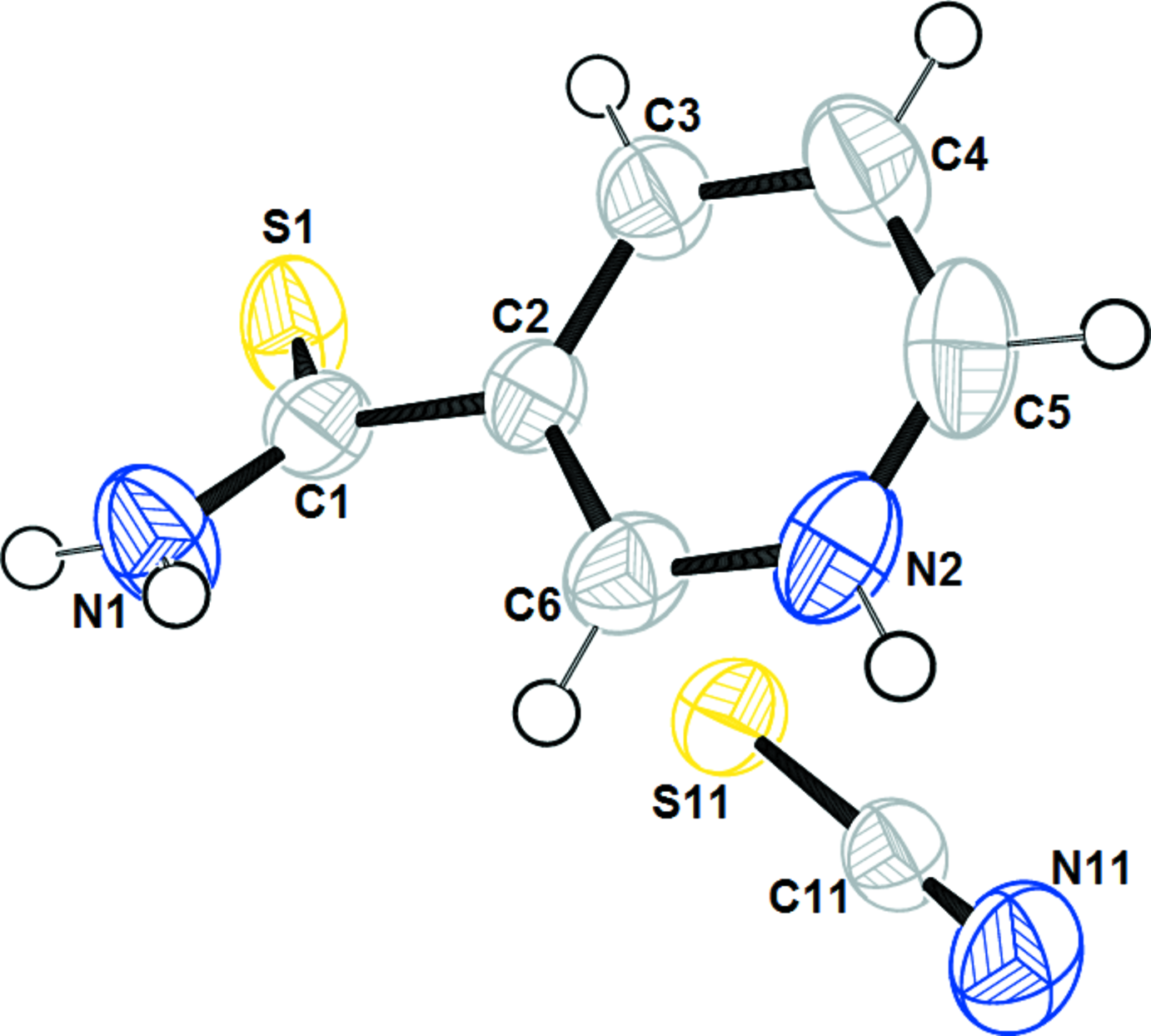

Supplement: Supplementary file 4 [file e-71-00o30-fig1.tif]

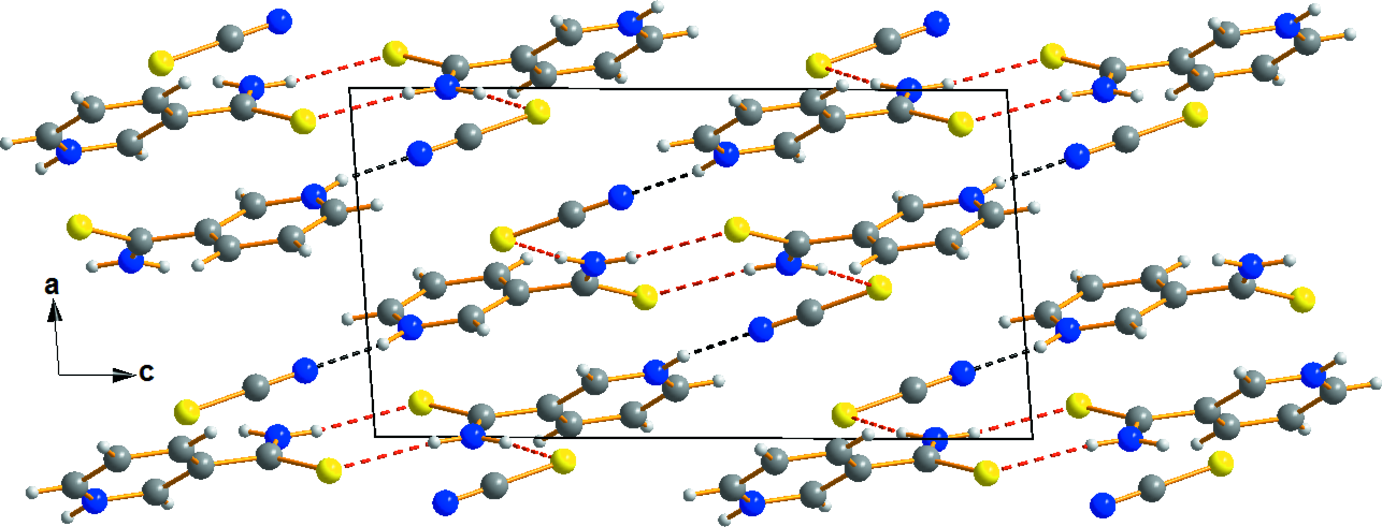

Supplement: Supplementary file 5 [file e-71-00o30-fig2.tif]
